# Aerobic Exercise Modulates Proteomic Profiles in Gastrocnemius Muscle of *db/db* Mice, Ameliorating Sarcopenia

**DOI:** 10.3390/life14030412

**Published:** 2024-03-20

**Authors:** Yen-Chun Huang, Monika Renuka Sanotra, Chi-Chang Huang, Yi-Ju Hsu, Chen-Chung Liao

**Affiliations:** 1 Graduate Institute of Sports Science, National Taiwan Sport University, Taoyuan 333325, Taiwan; 1091301@ntsu.edu.tw (Y.-C.H.); john5523@ntsu.edu.tw (C.-C.H.); ruby780202@ntsu.edu.tw (Y.-J.H.); 2 Marker Exploration Corporation, Taipei 112022, Taiwanmonika89m@gmail.com (M.R.S.); 3 Department of Cardiology, Taipei Veterans General Hospital, Taipei 112201, Taiwan; 4 Tajen University, Pingtung 907101, Taiwan; 5 Cancer and Immunology Research Center, National Yang Ming Chiao Tung University, Taipei 112304, Taiwan

**Keywords:** aerobic exercise, *db/db* mice, ECM remodeling, gastrocnemius muscle, metabolic pathways, muscle regeneration, proteomic profiling, T2DM-induced sarcopenia

## Abstract

Type-2 diabetes mellitus (T2DM)-induced sarcopenia is intertwined with diminished insulin sensitivity and extracellular matrix (ECM) remodeling in skeletal muscle and other organs. Physical activities such as aerobic exercise play a crucial role in regulating blood glucose levels, insulin sensitivity, metabolic pathways, oxidative stress, fibrosis, ECM remodeling, and muscle regeneration by modulating differentially expressed protein (DEP) levels. The objectives of our research were to investigate the effect of six weeks of aerobic exercise on the gastrocnemius and soleus muscle of *db/db* mice’s DEP levels compared to those of sedentary *db/db* mice. A total of eight *db/db* mice were divided into two groups (*n* = 4 per group), namely sedentary mice (SED) and exercise-trained mice (ET), of which the latter were subjected to six weeks of a moderate-intensity aerobic exercise intervention for five days per week. After the exercise intervention, biochemical tests, including analyses of blood glucose and HbA1c levels, were performed. Histological analysis using H & E staining on tissue was performed to compare morphological characters. Gastrocnemius and soleus muscles were dissected and processed for proteomic analysis. Data were provided and analyzed based on the DEPs using the label-free quantification (LFQ) algorithm. Functional enrichment analysis and Ingenuity Pathway Analysis (IPA) were employed as bioinformatics tools to elucidate the molecular mechanisms involved in the DEPs and disease progression. Significantly reduced blood glucose and HbA1c levels and an increased cross-sectional area (CSA) of gastrocnemius muscle fibers were seen in the ET group after the exercise interventions due to upregulations of metabolic pathways. Using proteomics data analysis, we found a significant decrease in COL1A1, COL4A2, ENG, and LAMA4 protein levels in the ET gastrocnemius, showing a significant improvement in fibrosis recovery, ECM remodeling, and muscle regeneration via the downregulation of the TGF-β signaling pathway. Upregulated metabolic pathways due to ET-regulated DEPs in the gastrocnemius indicated increased glucose metabolism, lipid metabolism, muscle regeneration, and insulin sensitivity, which play a crucial role in muscle regeneration and maintaining blood glucose and lipid levels. No significant changes were observed in the soleus muscle due to the type of exercise and muscle fiber composition. Our research suggests that engaging in six weeks of aerobic exercise may have a positive impact on the recovery of T2DM-induced sarcopenia, which might be a potential candidate for mitigation, prevention, and therapeutic treatment in the future.

## 1. Introduction

Persistent hyperglycemia and sarcopenia are major hallmarks of diabetes mellitus (DM), which is intertwined with metabolic disorders [1]. DM is caused by poor insulin secretion, decreased insulin sensitivity to peripheral effects, or a combination of these factors. It may cause organ damage, especially with respect to the skeletal muscles, kidneys, heart, brain, or eyes. According to the etiological classification of DM, type-2 diabetes mellitus (T2DM) is the most predominant, followed by type-1 diabetes mellitus (T1DM) and gestational diabetes (GDM). According to the International Diabetes Federation (IDF) 2021 report, a total of 537 million people live with diabetes, and it leads to over 4 million deaths per annum worldwide [2]. Data on complications of diabetes associated with microvascular (nephropathy—5.4%, retinopathy—2.3%, and neuropathy—1.9%) and macrovascular (coronary artery disease—7.4%, cerebrovascular disease—1.9%, peripheral artery disease—1.6%, and heart failure—2.3%) diseases were reported in Taiwan, and these complications could be responsible for lowering the quality of life of diabetic patients.

T2DM has modifiable (exercise, diet, body weight, body mass index, smoking, alcohol, blood pressure, lipids levels, sleep, and stress) and non-modifiable (genetics/family history, ethnicity, age, and gestational diabetes) risk factors [3]. Exercise can assist in managing body weight, body mass index, and consumption of stored energy in different organs (like skeletal muscle, the pancreas, the liver, and kidneys), thus controlling the progression of this pathology. A literature review shows that aerobic exercise can ameliorate T2DM manifestations by increasing insulin action, insulin sensitivity, mitochondrial density, oxidative enzyme numbers, blood vessel compliance and reactivity, lung function, immune function, and cardiac performance [4,5]. Running, walking, cycling, swimming, or jogging, examples of aerobic exercises, can treat sarcopenia without showing notable changes in body weight, which could be linked to improved insulin action and the regulation of protein synthesis. Previous research showed a significant reduction in blood glucose and HbA1c levels after an exercise intervention, as in our findings [6]. HbA1c not only offers a reliable indicator of chronic hyperglycemia but also has a significant influence on the probability of long-term diabetic complications.

According to our literature review, substantial impacts of physical activity on both mouse (C57BLKS/J) and human skeletal muscles have been extensively investigated under healthy conditions [7,8,9,10]. Alterations in metabolic pathways and tissue regeneration were remarkable in exercise groups compared to controls. Moreover, as prior investigations have reported, we identified similar differential proteins and canonical pathways (glycolysis, TCA cycle, fatty acid oxidation, etc.) that were involved in fast-twitch fibers more significantly than slow-twitch fibers [11]. Our preliminary findings show a noteworthy alteration in muscle loss in *db/db* mice as well as an improvement in the muscle density of gastrocnemius muscle after a six-week exercise intervention; but such observations were not made regarding the soleus, findings akin to those reported in previous research [12]. In addition, we also found differentially expressed proteins (DEPs) linked to fibrosis recovery and muscle regeneration via ECM remodeling. So far, comparative analyses demonstrating how aerobic exercise affects the skeletal muscle of *db/db* mice compared to that of sedentary controls have not been extensively investigated. Therefore, the purpose of this research was to establish *db/db* mouse models (sedentary and exercise-trained) that can mimic T2DM symptoms, like hyperglycemia and sarcopenia, and then investigate how aerobic exercise impacted fast- (gastrocnemius) and slow- (soleus) twitch muscle fibers. Our experiment explores the molecular mechanisms responsible for enhancing muscle function in diabetic mice through a *db/db* mouse exercise intervention.

## 2. Methods

### 2.1. Animal Model Design and Exercise Intervention

**Figure 1:**
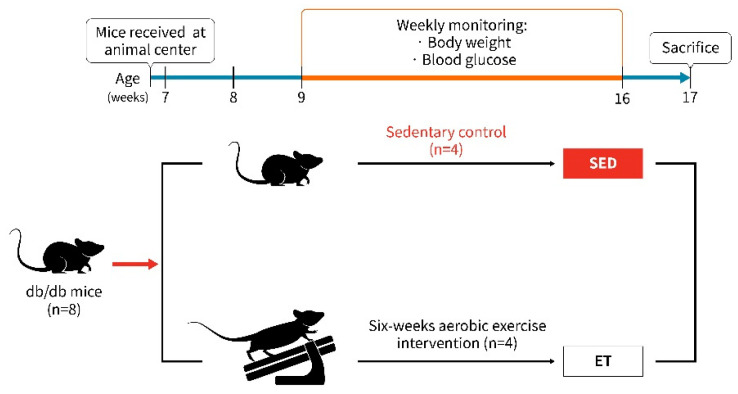
Illustration of animal model and research design. Eight-week-old male *db/db* mice (*n* = 8) were divided into two groups: the first group underwent six weeks of aerobic exercise training (ET, *n* = 4), and the other group was used as a sedentary (SED, *n* = 4) control.

Each experimental methodology for animal use in this research was approved by the National Taiwan Sport University Institutional Animal Care and Use Committee (IACUC-11003). The National Laboratory Animal Center provided male C57BLKS/J Iar-+Lepr^db^/+Lepr^db^ mice (*n* = 8) that were 7 weeks old (IAR, Tokyo, Japan). Water and a regular chow meal (no. 5001, PMI Nutrition International, Brentwood, MO, USA) were provided ad libitum. The mice were maintained in stable conditions with regulated humidity and a temperature range of 22 ± 2 °C in a 12-h light/dark cycle. The mice were divided into two groups (*n* = 4 per group): (1) *db/db* mice (C57BLKS/J Iar-+Lepr^db^/+Lepr^db^) that did not engage in exercise (sedentary-SED) and (2) *db/db* mice that underwent six weeks of aerobic exercise training (ET). Moderate-intensity aerobic exercise training includes frequency of treadmill (Model MK-680; Muromachi Kikai, Tokyo, Japan) use once a day, for five days a week, and for 10 min a day for six weeks. The starting speed was 10 m/min, and the weekly speed increased by 1 m/min until reaching 16 m/min. The slope gradually increased from a 5% to 10% incline after the third day. Body weight and blood glucose levels were measured twice a week. Forty-eight hours after the last exercise session, all the mice were sacrificed using the asphyxiation method (with 95% CO_2_), and their blood was collected immediately for biochemical tests. All tissues and organs, including gastrocnemius muscle and soleus muscle, were excised, rinsed in saline solution, and blotted dry for proteomics analysis. The total weights of the individual muscles were recorded, and the muscle tissue was frozen until further analysis. Our animal models and research design are well-explained in Figure 1.

### 2.2. Blood Glucose Levels

Before the blood glucose test, the mice underwent 16-h fasting. Then, their glucose levels were measured using the OneTouch^®^ UltraEasy^TM^ glucose monitor (Johnson, TX, USA) on a weekly basis [13]. A RAPIDIA auto HbA1c-L (FUJIREBIO Inc., Tokyo, Japan) system was used to measure the HbA1c levels in the mice’s blood before and after the exercise intervention.

### 2.3. Histological Analysis

The smaller section of gastrocnemius and soleus muscle from the SED and ET groups were fixed with formalin (10%) and embedded in paraffin, as previously described [10]. Thin tissue slices (4 µm) of the sections stained with hematoxylin and eosin (H & E) were digitalized using a Imager Z2 microscope (Carl Zeiss Microimaging, Göttingen, Germany) and TissueFAXS viewer-version: 7.1.6245.139 software (TissueFAXS Plus Scanning System, TissueGnostics, Vienna, Austria). The quantification of the cross-sectional area (CSA) of the muscle fibers was performed using ImageJ (version: 1.54d; Java 1.8.0_345) software.

### 2.4. Sample Preparation for Proteomics Analysis

Frozen gastrocnemius and soleus muscle samples of the mice were thawed and transferred into safe lock tubes (Eppendorf AG, Hamburg, Germany). Ceramic beads and cold lysis buffer were added to Eppendorf tubes containing muscle. A Bullet Blender^®^ homogenizer was used to prepare tissue lysate. After the completion of homogenization, tissue lysate was centrifuged at 15,000 rpm for 15 min, and then the supernatant was collected. The SMART digest kit Trypsin protocol (60109-103-B, Thermo Fisher Scientific, Waltham, MA, USA) was used for sample preparation via tryptic digestion at 72 °C for 4 h. Dithiothreitol (DTT) and iodoacetamide (IAA) were used for the reduction and alkylation of peptide digestion, respectively. Solid-phase extraction plates (SPE-Thermo Fisher Scientific) were used for sample clean-up. The final eluted peptide samples were lyophilized and stored at −30 °C.

### 2.5. Proteomic Analysis Using LC-MS/MS

Before performing an LC-MS/MS analysis of the lyophilized stored muscle samples, the zip-tip method was used for optimizing protein concentrations and desalting. Vacuum-dried samples were reconstituted in 10 μL of 0.1% formic acid. The nanoflow liquid chromatography system (U3000 nUHPLC, Thermo Fisher Scientific, Waltham, MA, USA) and a UPLC column (50 cm × 75 µm, 2.6 µm, Thermo Scientific™ Accucore™ 150 C18 column) were used for sample separation. Mobile phase A (0.1% formic acid in water) and mobile phase B (0.1% formic acid in acetonitrile) were used as a gradient flow and monitored from 2 to 95% B for 150 min at a flow rate of 0.300 μL/min. Separated peptides were ionized using a nano-spray at 1.975 V and then analyzed using an Orbitrap Fusion Lumos tandem mass spectrometer. Based on data-dependent acquisition (isolation width: 1.4 *m*/*z*) analysis in a positive ion mode, ions were captured, according to the signal intensity, within three seconds. Full-scan mode (with an *m*/*z* range of 375–1975), with a resolution of 30,000 at *m*/*z* 200, was used to collect peptide mass spectrometry data. If the charge state of the ions ranged from +2 to +6, then selected precursor peptide ions were stimulated with ultra-high purity (UHP) nitrogen gas with high-energy collision dissociation (HCD) to collect MS/MS fragment ions. PEAKS Studio 11 (Bioinformatics Solutions, Waterloo, ON, Canada) was used to analyze the collected MS/MS spectra raw data files using the Andromeda search engine and the UniProt mouse protein database, with the latter containing 17,097 protein sequences released in February 2022. Using proprietary software, a quantitative analysis of proteins using an MS label-free quantification (LFQ) algorithm based on the area under the curve (AUC) was performed to identify proteins when they matched with at least one unique peptide. The false discovery rate (FDR) was set as 1%. Results were normalized using the total ion chromatography (TIC) method, and the DEPs were assessed using expression fold and *t*-test values.

### 2.6. Bioinformatic Tools

The universal biological network roles of the DEPs involved in the gastrocnemius and soleus tissue lysate were evaluated using Ingenuity Pathway Analysis (IPA, Ingenuity Systems Inc., Redwood City, CA, USA, https://www.qiagenbioinformatics.com/products/ingenuitypathway-analysis accessed on 28 August 2023) software. IPA data processing was performed using Uniprot protein ID and expression fold changes of the DEPs, providing canonical pathways and biological function analysis. Fisher’s exact test was used to determine the significance −log(*p*-value) value using a threshold greater than 1.3. Functional enrichment analysis was performed using the David functional annotation tool to acquire data from the Kyoto Encyclopedia of genes and genomes (KEGG) pathways and Gene Ontology (GO) analysis, including biological processes (BPs), molecular function (MF), and cellular components (CCs) [14]. KEGG pathway analysis and GO analysis were performed using David bioinformatic tools after inserting the Uniprot protein ID of the upregulated and downregulated DEPs individually. A 1.1-fold change in expression and a *p*-value of less than 0.05 were used as the thresholds for identifying the DEPs between the two groups. Volcano plot analysis was performed using Python (version 3.12) software. The average fiber cross-sectional area (CSA, µm^2^) was quantified with reference to the H & E staining results for each group observed using ImageJ.

### 2.7. Statistical Analysis

An unpaired *t*-test (two-tailed *p*-value) was used for all data analyses, and the results were expressed as mean ± SD (*n* = 4 per group) using GraphPad Prism 8.4.3. All *p*-values less than 0.05 were considered statistically significant and denoted as * *p* < 0.05, ** *p* < 0.01, and *** *p* < 0.001.

## 3. Results

### 3.1. The Impact of Aerobic Exercise on Body Weight and Blood Glucose Levels

**Figure 2:**
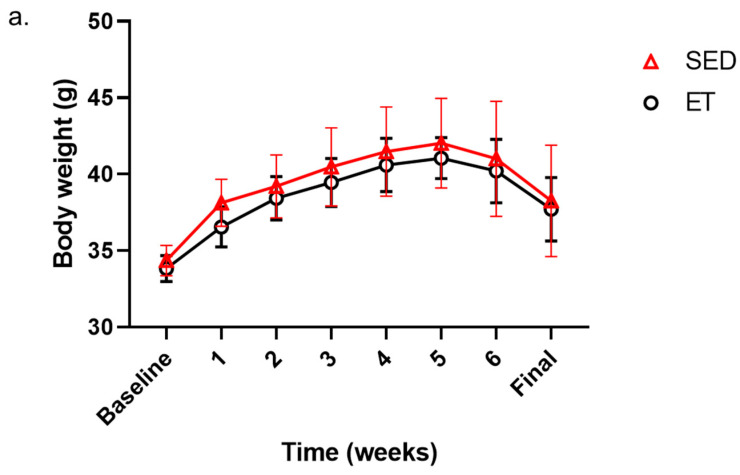

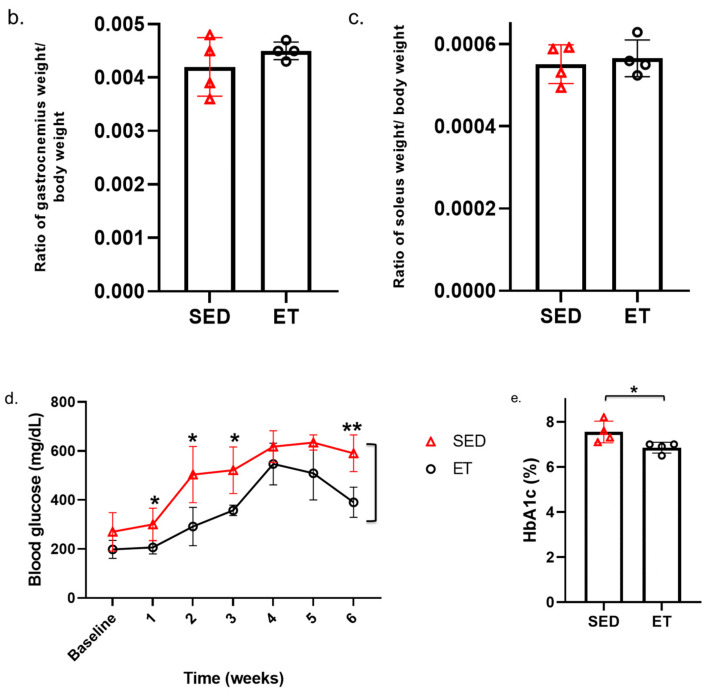
Impact of aerobic exercise on body weight, muscle weight, and blood glucose levels. (**a**) Changes in body weight (**a**) as well as the ratio of gastrocnemius muscle (**b**) and soleus muscle (**c**) weight to final body weight were recorded. Blood glucose levels (**d**) were monitored throughout the training sessions. HbA1c levels (**e**) were recorded before and after the exercise intervention. Body weight (**a**) and blood glucose levels (**d**) were recorded twice a week for six weeks in the SED and ET groups. Statistics are reported as mean ± SD for *n* = 4 mice per group, corresponding to the results obtained using an unpaired *t*-test (two-tailed *p*-value) applied to (**d**,**e**). The *p*-values are denoted as *p* < 0.05 *, <0.01 ** between the SED and ET mouse groups. ET—mouse group (*n* = 4) that was subjected to a six-week aerobic exercise intervention, HbA1c—glycated hemoglobin, SD—standard deviation, and SED—sedentary control group (*n* = 4).

The total body weight of the *db/db* mice in the SED and ET groups showed no notable change from the intervention period from week one to week six, although there was a slight decrease in the ET group compared to the SED group (Figure 2a). As anticipated, the ratio of gastrocnemius and soleus muscle weight to body weight in the SED and ET groups showed a moderate increase in the ET group compared to the SED group (Figure 2b,c).

We established the development of T2DM pathology in the *db/db* mouse models by validating glucose metabolism and skeletal muscle morphological changes in the SED and ET mouse groups. The HbA1c levels were measured before and after an exercise intervention, whereas blood glucose levels were monitored twice a week during the experimental period. Interestingly, we noted significantly decreased blood glucose levels at weeks one, two, three, and six in the ET group compared to these levels in the SED group during the exercise intervention (Figure 2d). However, higher levels of HbA1c (>6%) in both the SED and ET groups were obvious due to the inclusion of *db/db* mice in both groups (Figure 2e). However, HbA1c levels decreased significantly in the ET group (6.85 ± 0.24%) compared to the SED group (7.55 ± 0.48%) after six weeks of the exercise intervention (*p* < 0.05), as reported in previous studies [6].

### 3.2. The Impact of Aerobic Exercise on Skeletal Muscle Composition

**Figure 3:**
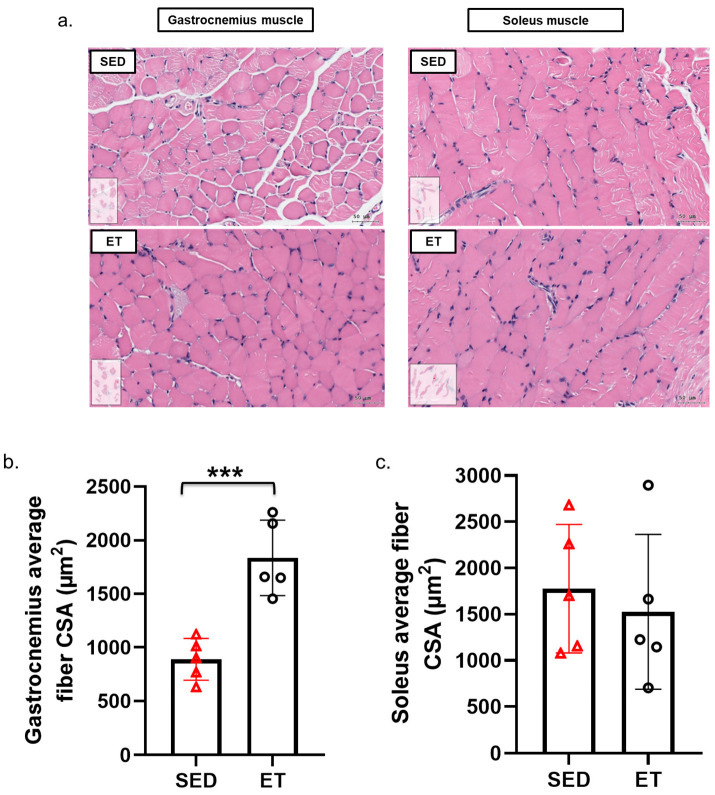
Impact of aerobic exercise on skeletal muscle composition. (**a**) Morphological changes in gastrocnemius and soleus muscle depicted use the hematoxylin and eosin (H & E) staining method (scale bar = 50 µm). Average fiber cross-sectional area (CSA, µm^2^) of the gastrocnemius (**b**) and soleus (**c**) quantified via H & E staining in each group (SED and ET) using ImageJ. Each group consisted of four mice. Statistics are reported as mean ± SD determined using an unpaired t-test (two-tailed *p*-value). The *p*-values are denoted as <0.001 *** between the SED and ET mouse groups. CSA—cross-sectional area, ET—mouse group (*n* = 4) that was subjected to a six-week aerobic exercise intervention, H & E—hematoxylin and eosin, and SED—sedentary control group (*n* = 4).

Hematoxylin (a deep-blue–purple stain applied to nucleic acids) and eosin (a pink stain applied to cytosol) (H & E) staining of the gastrocnemius and soleus muscle were used to validate T2DM-induced sarcopenia in the SED mice and recovery in the ET mice (Figure 3a). The results of the staining demonstrated a considerable reduction in the density and area of myofibers and increased fibrosis in the SED group, particularly in the gastrocnemius (type IIb) muscle. These results are consistent with previous research [12]. In contrast, the ET group showed that there was a significantly ameliorated effect of aerobic exercise on sarcopenia in terms of increased cell density, the area of myofibers, and fibrosis recovery in the gastrocnemius muscle based on the average fiber cross-sectional area (CSA, µm^2^) quantified in each group using ImageJ (Figure 3b). As per the literature review, we also found positive effects of aerobic exercise on gain in muscle mass, especially for muscle mainly composed of type II fibers, in T2DM patients [15,16]. However, no difference was seen in the soleus muscle due to its oxidative fiber type, propensity, and exercise capacity (Figure 3c). The HbA1c levels and histology suggested that modest muscle development may be attainable without changing body weight or muscle mass after a prolonged aerobic exercise intervention, a result that seems consistent with the earlier studies [12,17]. Based on these findings, we concluded that aerobic exercise contributes to sarcopenia recovery by improving the muscle cells’ composition and distribution and the morphological alterations of myofibers. Therefore, we developed a proteomics analysis method to understand further the molecular mechanism underlying T2DM-induced sarcopenia recovery in ET mice.

### 3.3. The Impact of Aerobic Exercise on the Proteomic Analysis of Gastrocnemius Muscle

**Figure 4:**
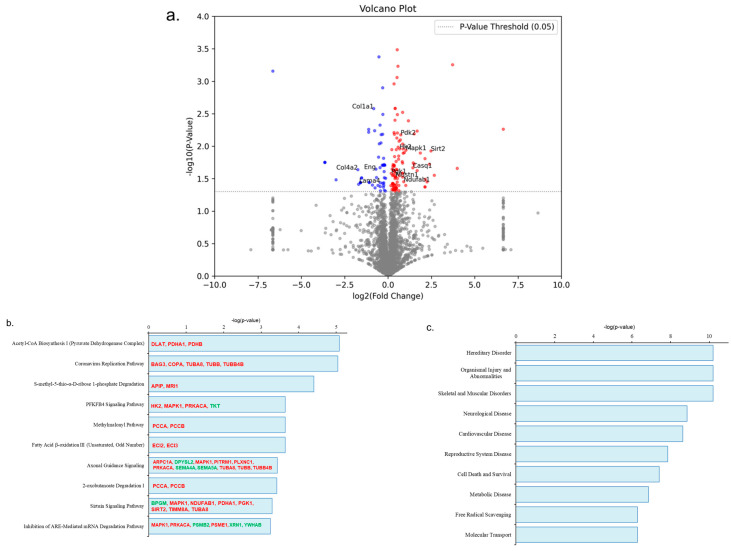

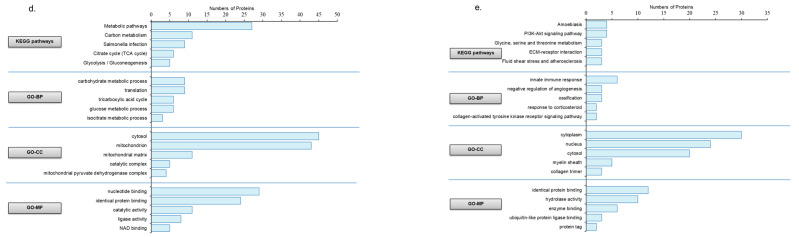
Impact of aerobic exercise on proteomic analysis of gastrocnemius muscle. (**a**) Illustration of proteomics data using a volcano plot. The x-axis and y-axis represent the ${log}_{2}$(FC) and ${-log}_{10}$(*p*-value) of protein levels, respectively. Red and blue circles represent significant upregulated and downregulated proteins, respectively. Grey circles indicate no differences in protein levels. The cutoff value of ${-log}_{10}$(*p*-value), set as 1.3, is represented by a horizontal dotted line (grey color). Fold change of DEPs greater than ±1.1 (${log}_{2}$(FC) > 0.14 or <0.14) were considered increased or decreased expression. The bar graphs show canonical pathways (**b**) and pathological biofunctions (**c**) modulated by DEPs in the gastrocnemius muscle of the ET group compared to the SED group. The canonical pathways minimum threshold of ${-log}_{10}$(*p*-value) was set as more than or equal to 1.3 (red bold font indicates upregulated DEPs and green bold font indicates downregulated DEPs). Functional enrichment analysis of upregulated DEPs (**d**) and downregulated DEPs (**e**) in the gastrocnemius muscle of ET mice including KEGG pathways and gene ontology (GO) analysis, including BP, CC, and MF. BP—biological processes, CC—cellular component, DEPs—differentially expressed proteins, ET—mouse group (*n* = 4) that was subjected to a six-week aerobic exercise intervention, FC—fold change, GO—gene ontology, KEGG—Kyoto encyclopedia of genes and genomes, MF—molecular function, and SED—sedentary control group (*n* = 4).

A total of 3426 proteins were found in the gastrocnemius muscle of the mice (Table S1). A volcano plot was used to highlight the significantly upregulated and downregulated proteins in the gastrocnemius muscle in the ET group compared to the SED group (Figure 4a). We found 155 differentially expressed proteins (DEPs) in the ET group after the six-week exercise intervention, including 103 upregulated and 52 downregulated DEPs (Table S2). To further investigate the biological roles of these DEPs, we applied an IPA analysis to gain further insight. Through IPA analysis, we found altered DEPs in the ET group that were involved in the regulation of the top ten canonical pathways (with a threshold of −log(*p*-value) ≥ 1.3), as indicated in (Figure 4b). The DEP changes in the ET gastrocnemius muscle significantly influenced skeletal and muscular disorder recovery in the ET group (Figure 4c). The structural composition of the gastrocnemius muscle (type IIb) is the primary reason for these notable changes in muscle damage recovery after exercise therapy [15]. Functional enrichment analysis, including KEGG pathways and GO analysis, revealed significant results regarding pathways and biological processes, like increased metabolism, insulin sensitivity, muscle regeneration, and angiogenesis, and decreased oxidative stress and fibrosis, similar to the IPA results (Figure 4d,e). Increased glucose metabolism (DLAT, HK2, MAPK1, PDHA1, PDHB, PDK2, PGK1, and SIRT2), fatty acid metabolism (ECI2, ECI3, NDUFAB1, PCCA, PCCB, and PDK2), muscle regeneration (APIP, ARPC1A, CASQ1, EHD2, MAPK1, MUSTN1, SIRT2, TUBA8, TUBB, and TUBB4B), and insulin sensitivity (HK2, MAPK1, and SIRT2) and decreased oxidative stress (MAPK1, MRI1, and SIRT2) were seen in the enrichment analysis of upregulated DEPs (Table S3). Furthermore, fibrosis recovery and muscle regeneration (COL1A1, COL4A2, ENG, and LAMA4) by transforming growth factor-beta (TGF-β) regulation were seen in the enrichment analysis of downregulated DEPs (Table S4) [17,18,19]. However, we also found insignificant downregulated TGF-β3 expression fold changes in the total protein list of the ET mice.

### 3.4. The Impact of Aerobic Exercise on Proteomic Analysis of Soleus Muscle

**Figure 5:**
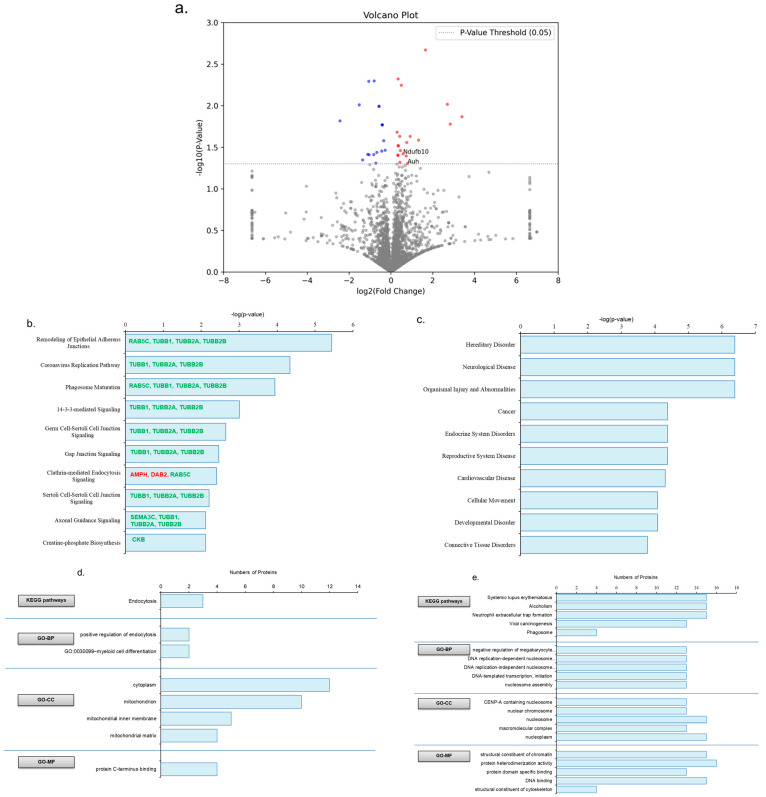
Impact of aerobic exercise on the proteomic analysis of soleus muscle. (**a**) Proteomics data illustrated by a volcano plot. The x-axis and y-axis represent the ${log}_{2}$(FC) and ${-log}_{10}$(*p*-value) of the protein levels, respectively. Red and blue circles represent significant upregulated and downregulated proteins, respectively. Grey circles indicate no differences in protein levels. The cutoff value of ${-log}_{10}$(*p*-value), set as 1.3, is represented by a horizontal dotted line (grey color). Fold changes in DEPs greater than ±1.1 (${log}_{2}$(FC) > 0.14 or <0.14) were considered as increased or decreased expression. The bar graphs show canonical pathways (**b**) and pathological biofunctions (**c**) modulated by DEPs in the soleus muscle of the ET group compared to the SED group. The canonical pathways minimum threshold of ${-log}_{10}$(*p*-value) was set as more than or equal to 1.3 (red bold font indicates upregulated DEPs and green bold font indicates downregulated DEPs). Functional enrichment analysis of upregulated DEPs (**d**) and downregulated DEPs (**e**) in the soleus muscle of ET mice including KEGG pathways and gene ontology (GO) analysis, including BP, CC, and MF. BP—biological processes, CC—cellular component, DEPs—differentially expressed proteins, ET—mouse group (*n* = 4) that was subjected to a six-week aerobic exercise intervention, FC—fold change, GO—gene ontology, KEG—Kyoto encyclopedia of genes and genomes, MF—molecular function, and SED—sedentary control group (*n* = 4).

On the other hand, 3662 proteins were found in the soleus muscle of the mice (Table S5). Unlike the data on the gastrocnemius muscle, the soleus muscle data did not include the multiple DEPs related to muscle regeneration. These results were likely obtained due to the latter’s composition of muscle fibers, i.e., type I, and the type of exercise engaged in, i.e., moderate-intensity aerobic exercise. Soleus muscle fibers show insignificant changes in mitochondrial functions and metabolic pathways after high-, moderate-, and low-capacity exercise for a short period [20]. As the volcano plot shows, there were fewer statistically significant DEPs in the mice that engaged in the training. (Figure 5a). Consequently, 38 DEPs were identified in the soleus muscle, including 20 upregulated and 18 downregulated DEPs (Table S6). As anticipated based on the IPA analysis, these DEPs did not appear to play any role in the top canonical pathways or biological processes relating to muscle regeneration, metabolism, or skeletal muscle. (Figure 5b,c). Although the KEGG pathways and GO analysis show the DEPs’ role, the corresponding results are not significantly similar to the IPA results (Figure 5d,e). In the KEGG pathways, the upregulated DEPs (AUH, MDH2, NDUFB10, PDXK, and SIRT5) show insignificant participation in metabolic pathways like fatty acid metabolism (AUH) and mitochondrial biogenesis (MDH2, NDUFB10, and SIRT5). Meanwhile, the biological process from the GO analysis included aerobic respiration (MDH2 and NDUFB10), which was not significantly affected (Tables S7 and S8). The proteomic data and morphological study seem consistent, providing evidence of no muscle regeneration in the soleus muscle after the exercise intervention.

## 4. Discussion

Our investigation focused on elucidating the ameliorating effects of aerobic exercise on type-2 diabetes mellitus (T2DM)-induced sarcopenia in *db/db* mice, specifically in the gastrocnemius and soleus muscles. Our proteomics analysis provided valuable insights into the molecular mechanisms underlying these effects.

Most proteomics research has been designed with respect to and conducted on skeletal muscles to investigate the impact of aerobic exercise on wild-type (C57BLKS/J) and high-fat-diet (HFD)-adherent mice compared to sedentary controls [8,9,10]. These HFD mice show increased obesity and insulin action impairment, much like *db/db* mice. These findings show that exercise-regulated proteins help to improve the insulin sensitivity of muscle and metabolic pathways and decrease fat mass by increasing glycolysis, TCA cycle, fatty acid catabolism, and fatty acid β-oxidation pathways, similar to our findings. Skeletal muscles are the primary sites for insulin-induced glucose and lipid metabolism, which are altered due to insulin resistance in obesity and T2DM pathology. Overeating and a physically inactive lifestyle trigger insulin resistance. So far, the molecular mechanisms involved in changing insulin sensitivity and the muscle mass of skeletal muscle in T2DM are still elusive.

Therefore, we decided to perform a proteomic analysis to understand the molecular mechanisms influencing the skeletal muscle of *db/db* mice after an exercise intervention. Our data show improved antioxidant levels, insulin action, metabolic pathways, muscle regeneration, and sarcopenia recovery in the gastrocnemius muscle of the ET mice. Our preliminary results reveal significantly decreased blood glucose and HbA1c levels and an increased cross-sectional area (CSA) of gastrocnemius muscle fibers in the ET group compared to the SED group. These results are consistent with the proteomics data and previous research [6,12]. However, no notable changes were seen in the soleus muscle mass and the CSA of the soleus muscle fiber, suggesting that the muscle adapted to exercise training largely due to a difference in muscle fiber composition. Therefore, we were interested in screening DEPs regulated by aerobic exercise that might have distinct physiological effects on the gastrocnemius muscle metabolism in response to ET compared to SED mice. We found 65 canonical pathways via an IPA analysis of ET-regulated proteins compared to those in the SED group and discussed the top ten most significant pathways that were more related to skeletal muscle regeneration. Daily physical activity can decrease fat mass and improve muscle insulin action in skeletal muscles through the induction of metabolic pathways [9]. Using IPA, KEGG, and GO analysis, we noticed 27 DEPs that overlapped between antioxidant levels, insulin action, metabolic pathways, muscle regeneration, and sarcopenia recovery. These potential protein candidates included twenty-seven DEPs, comprising a total of twenty-three upregulated and four downregulated DEPs. Glucose metabolism is enhanced in ET mice because of the increment in insulin sensitivity and glucose uptake and utilization in the skeletal muscle. Furthermore, lipid metabolism is increased in ET mice due to elevated fatty acid metabolism. These findings were validated through a functional enrichment analysis of upregulated DEPs involved in glycolysis, the TCA cycle, fatty acid catabolism, and fatty acid β-oxidation; additionally, downregulated DEPs, including COL1A1, COL4A2, ENG, and LAMA4, play a crucial role in muscle regeneration and the fibrosis recovery of skeletal muscle via ECM constituent clearance.

In ET mice, we observed that activated transcription regulators (COL4A2 and SIRT2), metabolic enzymes (PCCA and PCCB), and serine–threonine kinases (MAPK1) in skeletal muscles improved the metabolic pathways and insulin action [8]. Increased SIRT2 is crucial for muscle regeneration, the cell cycle, metabolic pathways, antioxidants, and insulin sensitivity [21,22,23]. The roles of SIRT2 in the sirtuin signaling pathway is (*p* < 0.05) according to IPA, the metabolic pathways are (*p* < 0.0001) according to the KEGG pathways, and the negative regulation of ROS metabolic process is (*p* < 0.01) according to GO-BP were validated in this research. Interestingly, a significant upregulation of MAPK1 was found in the ET mice compared to the SED mice, and MAPK1 participated in pathways and biological functions similar to those for SIRT2. The biological processes (BP) from the GO (GO-BP) analysis show significantly increased fatty acid beta-oxidation pathways due to upregulated DEPs, including ECI2, ECI3, DECR1, and TUBA8. EHD2, which is required for myoblast fusion, and these levels increased significantly in the ET mouse group compared to the SED group [24]. The gastrocnemius muscle showed higher levels of CASQ1 and CASQ2, which are required for muscle regeneration, in the ET mice compared to those in the SED mice. Interestingly, we observed a significant increase in CASQ1, which was approximately two times the CASQ2 levels in the ET gastrocnemius muscle. Calsequestrin 1 and 2 (CASQ1 and CASQ2) proteins are essential for regulating calcium release, muscle contraction, and the proliferation of muscle fibers, as previously described [11,25,26]. These findings were reported in CASQ1-null mice compared to a wild-type (C57BLKS/J) mouse model. Deficiency of these proteins significantly affects the structure of skeletal muscles and sarcoplasmic reticulum (SR) development via controlling calcium release. Generally, CASQ1 and CASQ2 levels are highly expressed in fast- (gastrocnemius) and slow- (soleus) twitch muscles, respectively. The exercise-training-upregulated proteins (HK2, NDUFAB1, PDK2, and SIRT2) observed in the IPA and GO-BP analyses are involved in the cellular response to the negative regulation of ROS [27]. Additionally, increased levels of ROS are cleared by antioxidant proteins (CCS), which help maintain muscle structure, function, and metabolism in healthy and diseased conditions. Upregulated MAPK1 and CCS proteins in the ET mice were responsible for redox homeostasis.

Functional enrichment analysis of downregulated DEPs, including KEGG pathways and GO-BP analysis, showed results consistent with the IPA data. The GO-BP analysis indicated that there was a negative effect on the regulation of angiogenesis (PEDF, COL4A2, and SEM4A) in the gastrocnemius muscle of the ET mice. These three downregulated DEPs, after aerobic exercise intervention, can promote angiogenesis by improving insulin sensitivity and capillary density [28]. In addition, a decreased response to corticosteroid hormone (*p* < 0.01) was also seen in the ET compared to the SED group, including, with respect to, COL1A1 and ENG [29]. Overall, these downregulated DEPs enhance muscle regeneration through ECM-receptor interaction (COL1A1, COL4A2, and LAMA4), increased angiogenesis, and decreased response to corticosteroids in ET mice for the structural maintenance of fast-twitch (type-IIb) muscle fiber like that of the gastrocnemius. These proteins play a crucial role in ECM remodeling by clearing collagens and laminins after an exercise intervention [30]. In contrast, LAMA4 and COL4A2 levels increase significantly in diabetic conditions, causing forms of muscle dystrophy like reduced muscle cell density and area, necrosis, and fibrosis. The decreased collagen levels in the ET mice positively correlated with increased myonuclear per fiber and average gastrocnemius cross-sectional area (CSA), reduced fibrosis, and improved angiogenesis. Results obtained from the H & E staining and proteomic analysis of ET mice provide evidence of increased muscle regeneration without notable changes in body weight [12]. The H & E slides show increased myofiber numbers, area, and density. They also indicate fibrosis recovery in the ET mice compared to the SED group, consistent with the altered DEPs related to fibrosis recovery and angiogenesis from proteomic analysis.

**Figure 6:**
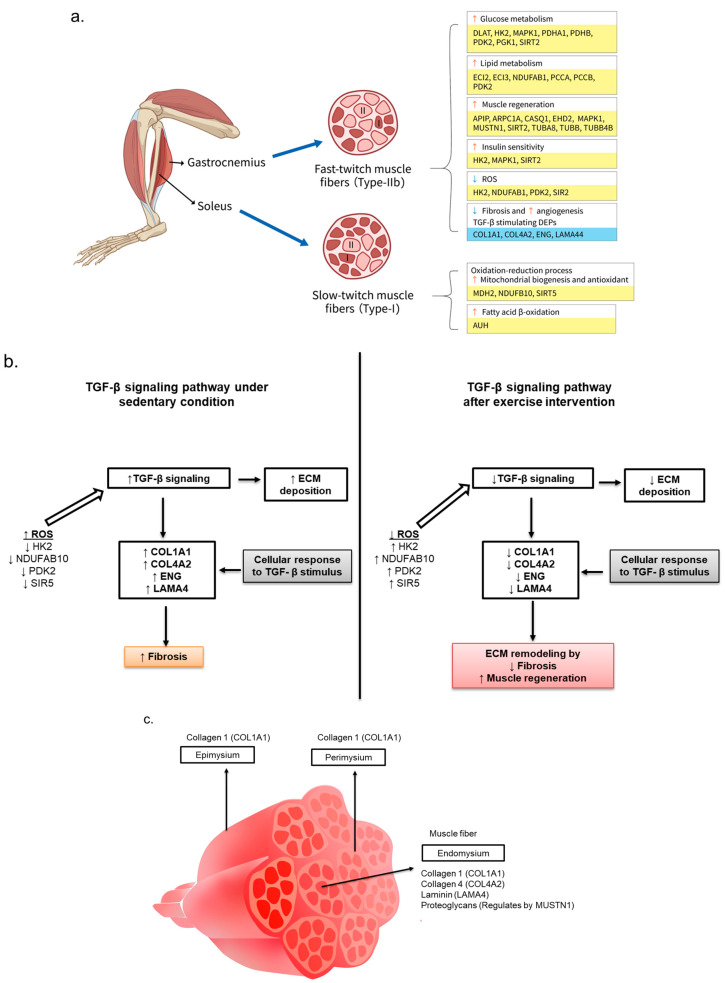
Summary of proteomics analysis of gastrocnemius and soleus muscle in the ET group. (**a**) Exercise-training-regulated proteins’ role in improving metabolic pathways (glucose and lipids), muscle regeneration, insulin sensitivity, antioxidant levels, fibrosis, and angiogenesis. Red and pink muscle fiber types are denoted by I and II, respectively. Upregulated proteins are highlighted with a yellow background, while downregulated proteins are highlighted with a blue background. The anticipated increases and decreases in biofunction are represented by the orange and blue arrows pointing upward and downward, respectively. (**b**) The ameliorating effect of aerobic exercise on T2DM-induced sarcopenia through ECM remodeling in the gastrocnemius muscle via a TGF-β signaling pathway. Increases and decreases in DEP levels are represented by the black arrows pointing upward and downward, respectively. (**c**) Localization of DEPs linked to muscle regeneration in gastrocnemius muscle. DEPs—differentially expressed proteins, ECM—extracellular matrix, ET—mouse group (*n* = 4) that was subjected to a six-week aerobic exercise intervention, ROS—reactive oxygen species, TGF-β—transforming growth factor-beta, and SED—sedentary control group (*n* = 4).

A proteomic analysis summary of the gastrocnemius muscle in the ET group compared to that in the SED group revealed improved metabolic pathways, insulin sensitivity, muscle regeneration, ROS clearance, angiogenesis, and fibrosis recovery (Figure 6a). Mainly, DEPs such as COL1A1, COL4A2, ENG, LAMA4, and MUSTN1 (abundantly expressed in the endomysium of muscle fiber) play crucial roles in the muscle regeneration and fibrosis recovery of skeletal muscles via ECM remodeling, as shown in the GO-BP analysis [19,31,32]. Accumulation of ECM deposition and tissue fibrosis can be promoted by increased levels of collagens, laminins, and endoglin due to the stimulation of the TGF-β signaling pathway under oxidative stress (Figure 6b,c). Increased ECM deposition and decreased clearance could be common with aging or pathological disorders like T2DM. Endoglin and collagen-4 regulate the activated TGF-β levels, which could be the possible reason for the lack of a notable change in TGF-β levels, similar to our data. Therefore, although with an insignificant decrease in TGF-β3 of ET mice, we still observed significantly decreased levels of collagens (1 and 4), laminins (LAMA4), and endoglin (ENG) for initiating muscle fiber differentiation and proliferation to allow recovery from tissue fibrosis and sarcopenia in *db/db* mice. The mechanism underlying the interaction between downregulated proteins and TGF-β signaling pathways in renal or cardiac muscle fibrosis was illustrated using cell models, e.g., NRK-49F and C2C12 myoblasts, in previous research [33,34]. We identified DEPs (COL1A1, COL4A2, ENG, and LAMA4) and canonical pathways (angiogenesis and ECM remodeling) associated with fast-twitch fibers similar to those found in previous studies. Additionally, the reduced oxidative stress seen in the ET mice was induced by upregulated DEPs (HK2, NDUFAB10, PDK2, and SIRT2), inhibiting ROS production as well as TGF-β stimulation. Our findings revealing the positive impact of aerobic exercise on sarcopenia recovery via ECM remodeling could be used as a potential therapeutic treatment to improve the quality of life of diabetic patients after clinical studies. While it is widely known that different exercises lead to distinct muscle adaptations, our treadmill-based training of diabetic mice, typically considered aerobic, yielded unexpected results. Contrary to expectations, the gastrocnemius muscle, not the soleus muscle, exhibited proteomic changes associated with aerobic exercise. Moreover, the cross-sectional analysis revealed an increase in cross-sectional area in the gastrocnemius muscle, suggesting reduced muscle loss compared to the sedentary control group. These observations prompt considerations regarding the interplay between exercise intensity and muscle-specific responses in diabetic animal models e.g., *db*/*db* mice. Further exploration is needed to unravel the underlying mechanisms and potential implications of these unanticipated findings in our study. Muscle fibers are divided into type I (oxidative) and type II (glycolytic) muscle fibers [35]. Muscle fiber transformation depends on exercise intensity and muscle fiber propensity. Although aerobic exercise is known to have a significant impact on soleus muscle mass and protein levels, this effect was not observed in our findings [36]. Previous studies also show a significant response of aerobic exercise (for 8 and 13 weeks) on type II muscle fibers but not type I muscle fibers. Endurance training induces muscle fiber switch towards oxidative pathways, but the soleus muscle is comprised predominantly of type I fibers, resulting in a less pronounced shift [37]. Additionally, the fact that the mitochondrial function increased significantly by high-intensity exercise compared to moderate- or low-intensity aerobic exercise shows the reproducibility of our findings [20]. This study also reported that there were no differences seen in the metabolic profile of the soleus muscle in 9- and 18-month-old rats after high- and low-intensity exercise training. This indicates that some muscles have a higher propensity for fiber-type shifting compared to others. It may be possible that the soleus muscle requires higher exercise intensity to exhibit a response. The use of an animal model is a limitation of our study. Even though the metabolic processes and regulation of these processes are identical between humans and mice, there are still significant differences, e.g., in terms of metabolic rate. It is, therefore, unclear whether T2DM or exercise control the same proteins or whether these proteins control various metabolic pathways in human muscle tissue.

In conclusion, T2DM and aerobic exercise significantly impact skeletal muscles. We reported potential DEP candidates regulated by aerobic exercise in the gastrocnemius muscle of *db/db* mice, showing an ameliorating effect on T2DM-induced sarcopenia. The occurrence of muscle regeneration due to aerobic exercise was supported by the quantification of downregulated DEPs (COL1A1, COL4A2, ENG, and LAMA4) in gastrocnemius muscle. These DEPs are involved in ECM remodeling because of the improved insulin sensitivity and metabolic pathways in the ET group. Nevertheless, because of our findings, this can now be dealt with in focused subsequent clinical research investigations, which our outlook has guided. The illustration of this novel mechanism could help in the use of aerobic exercise as a pharmacological intervention to improve the quality of life among T2DM patients.

## Data Availability

The data presented in this study are available on reasonable request from the corresponding author.

## Supplementary Materials

The following supporting information can be downloaded at: https://www.mdpi.com/article/10.3390/life14030412/s1, Table S1 Total proteins (3426) quantified in gastrocnemius muscle of ET mice compared to SED after proteomic analysis. Table S2 List of DEPs (155) in the gastrocnemius muscle of ET group compared to SED after proteomic analysis. The fold change represented in green and red bold fonts show downregulated and upregulated DEPs respectively. Table S3 Functional enrichment analysis of upregulated DEPs in the gastrocnemius muscle of ET compared to SED mice. Table S4 Functional enrichment analysis of downregulated DEPs in the gastrocnemius muscle of ET mice compared to SED mice. Table S5 Total proteins (3662) quantified in soleus muscle of ET mice compared to SED after proteomic analysis. Table S6 List of DEPs (38) in the soleus muscle of ET group compared to SED after proteomic analysis. The fold change represented in green and red bold fonts show downregulated and upregulated DEPs respectively. Table S7 Functional enrichment analysis of upregulated DEPs in the soleus muscle of ET compared to SED mice. Table S8 Functional enrichment analysis of downregulated DEPs in the soleus muscle of ET mice compared to SED mice.
